# Pleural effusion in difficult weaning from mechanical ventilation

**DOI:** 10.1186/2197-425X-3-S1-A92

**Published:** 2015-10-01

**Authors:** M Dres, D Roux, T Pham, M Fartoukh, JD Ricard, A Demoule

**Affiliations:** UPMC Université Paris 06, INSERM, UMRS 1158 Neurophysiologie Respiratoire Expérimentale et Clinique, Sorbonne Universités, Paris, France; Hopital Pitie Salpetriere, Assistance Publique Hôpitaux de Paris, Respiratory and Critical Care Department, Paris, France; Assistance Publique - Hopitaux de Paris, Louis Mourier Hospital, Critical Care Department, Colombes, France; Assistance Publique - Hopitaux de Paris, Tenon Hospital, Critical Care Department, Paris, France

## Introduction

Pleural effusions (PE) are common in intensive care unit (ICU) patients, especially in patients under mechanically ventilation. Since PE can alter gas exchanges, one could hypothesize that PE could play a role in the outcome of weaning from mechanical ventilation.

However, no study has yet reported the incidence and characteristics of PE in the specific context of weaning failure.

## Objectives

To describe the incidence of pleural effusion in patients who failed a first spontaneous breathing trial (SBT) and to describe the characteristics of these patients.

## Methods

We conducted a prospective observational study in three medical ICU. All mechanically ventilated patients were screened daily. In patients who failed their first SBT, a pleural ultrasonography was performed and the presence of PE was qualitatively quantified according to a 4-steps classification: 0: no PE; 1: small PE; 2: moderate PE and 3: large PE. In addition the main clinical characteristics of patients were collected. For statistical analysis, patients with classes 0 and 1 were regrouped, as were patients with classes 2 and 3.

## Results

From November 2014 to March 2015, 336 patients were screened and 56 (17%) failed their first SBT. Among them, 28 patients (50%) had no PE, 18 patients (32%) had small PE (left or right), 8 (14%) patients had moderate PE and 2 (4%) patients had large PE.

Patients with moderate and large PE stayed longer in ICU (19 ± 7 vs. 9 ± 7 days, p < 0.01) and had a longer duration of mechanical ventilation (14 ± 9 vs. 24 ± 12 days, p < 0.01) as compared with patients without PE and with small PE.

Patients with moderate or large PE and patients without PE or with small PE shared similar characteristics at admission (age: 66 ± 14 and 61 ± 15 and SAPS 2: 55 ± 18 53 ± 15, p > 0.05).

Shock as the cause of initiation of mechanical ventilation was more frequent in patients with moderate and large PE as compared to patients without and small PE (40% vs. 4%, p < 0.01).

Only one patient with a large PE had a pleural evacuation but without clinical improvement.

## Conclusions

Significant pleural effusion is often detected in difficult to wean patients (18%), and is associated with shock state as the reason for mechanical ventilation. The interest of pleural drainage in this population deserves further studies.

